# A roadmap to reduce the incidence and mortality of breast cancer by rethinking our approach to women’s health

**DOI:** 10.1007/s10549-024-07522-4

**Published:** 2024-11-12

**Authors:** Katherine Leggat-Barr, Douglas Yee, Erin Duralde, Caroline Hodge, Virginia Borges, Molly Baxter, Jessica Valdez, Tamandra Morgan, Judy Garber, Laura Esserman

**Affiliations:** 1 Tufts Medical School, Boston, MA USA; 2 Masonic Cancer Center Minneapolis, University of Minnesota, Minneapolis, MN USA; 3 One Medical, Los Angeles, CA USA; 4 Department of Surgery, University of California, San Francisco, San Francisco, CA USA; 5 Division of Medical Oncology, University of Colorado Cancer Center, Aurora, CO USA; 6 Johns Hopkins Medical School, Baltimore, MD USA; 7 Dana Farber Cancer Institute, Boston, MA USA; 8 Alfred A de Lorimier Endowed Chair in General Surgery, 1825 4th St, 3rd Floor, San Francisco, CA 94158 USA

**Keywords:** Breast cancer, Breast cancer risk, Hormonal interventions

## Abstract

Despite progress, breast cancer remains the most feared disease among women. In the USA alone, the incidence is now almost 300,000 new cancers per year, a rate that has nearly doubled in the last 30 years. Most women survive, but over 40,000 women a year still die of their disease [99]. It is the most diagnosed cancer among women and the second leading cause of cancer death. Important disparities exist in breast cancer outcomes among African American women, where women die of breast cancer at higher rates, are diagnosed younger, and at a more advanced stage. We are proposing a radical shift in our thinking about breast cancer prevention with an aspiration to dramatically lower breast cancer incidence. Most breast cancers are driven by steroid hormones. Throughout the life course, women are offered an array of hormonal treatments for menstrual cycle control, family planning, in vitro fertilization, postpartum weaning, and menopausal symptom management. There are mixed data on the extent to which each of these may contribute to increased or decreased risk for breast cancer. These endocrine manipulations could represent a great opportunity to potentially reduce breast cancer incidence and improve quality of life for survivors. To date, they have not been designed to explicitly reduce breast cancer risk. A new holistic approach will require scientists, drug developers, breast oncologists, obstetricians, gynecologists, endocrinologists, radiologists, and family medicine/internists to work together toward the common goal of reducing breast cancer risk while addressing other critical issues in women’s health.

## Introduction

A critical and yet untapped approach to reducing breast cancer incidence could come from redesigning the routine hormonal interventions most women use at some point of their lives for symptom amelioration or cycle control, as opportunities to improve breast health. These medical interventions can support broader efforts that are also needed to promote the healthy environments and lifestyles associated with greater health span.

We use endocrine modulation for cycle control, contraception, fertility preservation, and menopause symptom management, as well as to treat breast cancer and prevent recurrence. Existing methods are often at odds with each other. For example, the most common forms of menopausal hormone therapy treat symptoms well but increase the risk of breast cancer by a small amount [1]. Agents such as tamoxifen and aromatase inhibitors reduce breast cancer recurrence and incidence, but cause side effects, though dose reductions in tamoxifen have now been found effective for prevention with minimal side effects. Could a more nuanced and targeted approach to endocrine modulation achieve women's health aims while reducing the risk of breast cancer? This enticing opportunity is a clarion call for urgent investment in research and development to incorporate breast cancer interception and prevention in all aspects of women’s health.

By bringing all the relevant fields together, we can reconceptualize our approach to women’s health. Starting from the lens of reducing the incidence of breast cancer, we can then broaden the perspective to improve options for women over the course of the many junctures where hormonal and lifestyle interventions are often considered. Here, we will:

1. Review advances in breast cancer tumor profiling and personalization of therapy;
2. Reimagine screening as part of a larger effort to reduce risk, where risk assessment can personalize both risk reduction and screening;
3. Review opportunities to incorporate risk reduction strategies into hormonal product design and management, as well as strategies to shorten the product development cycle;
4. Identify opportunities to take lessons from breast cancer prevention back full circle to safely improve the quality of life of survivors of breast cancer;
5. Work to reduce misinformation about hormonal products and increase investment in generating relevant data to advance the field and our stated goals of reducing breast cancer incidence while also addressing hormonal management needs.
6. Convene an interdisciplinary group of clinicians and scientists as well as regulators and drug and investors to design, develop and test new products that will work for symptom amelioration and breast cancer prevention and envision a new women's health initiative.

**Fig. 1:**
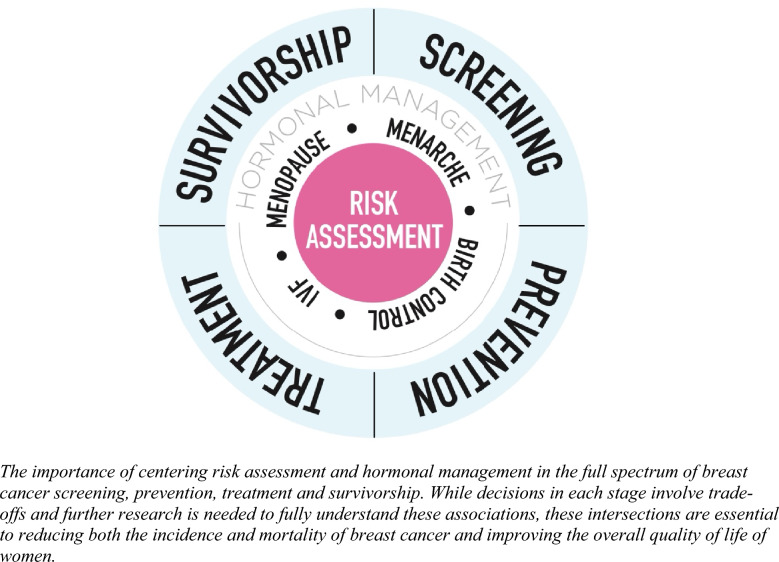
Opportunities to reduce the incidence and mortality of breast cancer with a holistic approach to women’s health

Cross-disciplinary collaboration is necessary to achieve such a paradigm shift. Our goal is to engage all women’s health providers (primary care, OBGYN, endocrinology, breast oncologists), scientists pharmaceutical developers, regulatory agencies, and advocates in this effort. Breast cancer is not one disease—we do not treat it that way and we should not screen for it as if it is one disease or as if all women have the same risk. By the same token, all estrogens and progestogens are not equal and the products that we tested 20 years ago that were shown to increase the risk of breast cancer have also changed. The breast oncology community should both welcome and lead research that can reduce risk of recurrence and yet also reduce side effects that could improve the lives of cancer survivors. Together, we can better inform ourselves and our patients. We need continued and committed innovation to better design hormonal products that are designed to both reduce breast cancer risk and alleviate the litany of adverse symptoms women and survivors of breast cancer experience from current risk-reducing interventions, as well as hormonal changes across the life course (Fig. 1).

We can take inspiration from our colleagues in cardiology, where a concerted effort to bring down the death rate from cardiac disease transitioned into routine risk assessment and management as part of primary care. The landmark 1945 Framingham study was the start of this revolution in cardiac care and provides a useful reminder of what can be accomplished: connecting risk assessment to modifiable risk factors, like blood pressure and cholesterol, transformed the field. These surrogate endpoints led to the development of risk-reducing agents and lifestyle intervention programs applied in the primary care setting, resulting in lower cardiovascular disease mortality. Over the past 70 years, risk tools and intermediate markers have been refined to great effect. The risk of dying from coronary heart disease has now fallen 60% since the 1960s, largely because of these advances in prevention [2].

A similar prevention path is possible for breast cancer. The challenge in breast cancer is that multiple commonly used hormonal medications used to improve women’s quality of life may increase breast cancer risk, making it more challenging to achieve our goal. However, as we better understand the ecosystem of estrogen, progesterone, testosterone, and glucocorticoid receptors and explicitly look for opportunities to strike the right balance, we just might achieve both a revolution in prevention and improve the quality of life of women, including survivors of breast cancer. In short, we should aspire to radically alter the negative impact of breast cancer by adopting a longitudinal approach that incorporates risk reduction and symptom management across the course of a woman's life.

### Section 1: we have made significant progress in better understanding tumor biology and tailoring subsequent treatments

In the past several decades, enormous progress has been made in characterizing the biology and heterogeneity of breast cancer. There are many types of breast cancer, spanning a range of aggressiveness and recurrence risk. At its most basic, tumors are classified as hormone receptor (HR) negative/positive and human epidermal growth factor receptor 2 (HER2) receptor-positive/negative. Based on both natural history and long-term trial outcomes, HR-positive tumors are generally considered slower growing, but have a risk of distant recurrence that extends for many years; HR-negative and HER2 + cancers are generally faster growing, their recurrence risk largely confined to the 5 years after diagnosis [3, 4].

Improved tumor classification beyond HR and HER2 [5] has led to the development of targeted therapies, including immune-directed therapies in combination with antibody–drug conjugates or chemotherapy for fast-growing immune-driven tumors (HR + and HR −) [6–8]. These targeted therapies have revolutionized how we treat breast cancer, leading to greater response and higher survival rates. We have learned that some HR-positive tumors are biologically more like triple-negative tumors. These advances may provide important information about how to develop subtype risk assessment tools and generate prevention interventions and manage the treatment of survivors of breast cancer.

One of the important advances that has accelerated progress in finding better therapies is the development of early endpoints for trials. When systemic therapy is sequenced before surgery (neoadjuvant) for fast-growing (molecularly high risk) stage 2 and 3 cancers, response to treatment can be measured using MRI and pathology, and these endpoints predict 3- and 5-year survival, with a hazard rate of 0.18 [9]. Neoadjuvant therapy allows a more personalized treatment approach, and the ability to reduce the extent of surgery, radiation and adjuvant therapy. Using the intact tumor to measure response, we provide patients and their clinicians the ability to forecast how they will do at 3 to 6 months, rather than waiting for 3–5 years after all patients have completed accrual to the trial [10]. Over the last 15 years, the pCR rates have gotten higher, presaging the reduction in mortality from aggressive breast cancer. Patients with hormone-responsive tumors need different therapeutic approaches than immune-driven tumors.

Endocrine therapy is the main intervention for hormone-positive tumors. Selective endocrine receptor modulators like tamoxifen are given in premenopausal women and aromatase inhibitors in postmenopausal women, largely due to concerns about small increases in uterine cancer (1.5% additional risk) and blood clots in older women, though lower doses of tamoxifen may abrogate that risk [11]. For women with aggressive hormone-positive tumors, ovarian suppression and aromatase inhibitors (lowering total estrogen exposure) have significantly better outcomes, leading to concerns about any endocrine replacement in this setting [12–14].

Tumor biology should absolutely inform our approach to screening and prevention and early endpoints accelerate the pace of learning. Germline risk factors, including pathogenic mutations in specific genes and Polygenic Risk Scores (PRS) from the many genes we inherit, have emerged as a risk predictors for specific types of cancer [15, 16]. Subtype-specific PRS could therefore help us to identify women who would specifically benefit from risk-reducing medications and interventions. Classes of drugs that target BRCA 1 and 2 tumors could eventually be developed for prevention in mutation carriers. Boosting immunity or vaccines could be considered for those at risk for immune-driven tumors. The key is to better understand who is at risk for what type of tumor and find ways to measure impact early.

### Section 2: reimagine screening as part of a bigger effort to reduce risk, starting with risk assessment first, and using this to personalize both risk reduction and screening

#### Our current screening paradigm

Currently, our breast screening program in the USA is a one-size-fits-all approach, assuming that all women are at equal risk of developing cancer, except for those with an inherited mutation where intensive screening is recommended, including annual mammography alternating every 6 months with annual MRI. Despite constantly shifting guidelines, annual mammography starting at 40 has remained the standard of care in the USA for over four decades regardless of one’s personal risk [17–20]. One of the shortcomings of this screening approach is that most aggressive cancers are not caught early [21]. Given we know breast cancers are not all the same and that risk factors differ among women, it does not make sense to screen as if everyone is the same. As with treatment, a more personalized approach to screening may be just as safe and more beneficial [19]. Trials of these new approaches are in progress [19, 22].

For women at very high risk, especially in the setting of extremely dense breast tissue, contrast-based imaging is clearly superior [23]. However, breast density should not be the only criteria for determining who receives supplemental screening, as 50% of women have BIRADS C and D density [24]. Contrast-based imaging can cause harm because it is expensive, as it has a large copay for most women, and has a high false-positive rate which can be stressful. There is clearly room for improvement in how we identify who is most likely to benefit from MRI screening. For example, even though guidelines recommend that women with a lifetime risk of 20% receive MRI screening, there is little data to support this improves outcomes [25]. Fortunately, other alternatives are in development that may be better for higher risk women. These include contrast enhanced mammography and fast MRI. These should reduce the cost and time and possibly false-positives and should be studied. As well, new imaging-based tools may be able to find the 10% of women with dense breast tissue that truly have high risk and warrant additional screening [26].

#### Tools for predicting breast cancer risk

Risk assessment has advanced significantly in the past decade. Several risk prediction models are available. Most include clinical risk factors like onset of menses, parity, family history, weight, and breast biopsy history. Many include breast density [27, 28] and some include rare coding variants in susceptibility genes and polygenic risk scores (PRS) [29]. Also emerging are artificial intelligence models that use mammographic features, including density, to predict risk [30, 31]. Breast cancer risk calculation, however, is not a routine part of clinical practice. Nor have the mammographic AI risk tools been integrated with the PRS and other risk models. There is much work to be done and an opportunity to significantly improve forecasting of both lifetime and short-term risk. Risk assessment, and the identification of high-risk individuals, is critical to designing effective, targeted interventions to prevent breast cancer.

#### Studies of personalized risk assessment and current gaps

The WISDOM study (Women Informed to Screen Depending on Measures of risk) uses a tripartite risk assessment, assessing participants' risk by the Breast Cancer Surveillance Consortium model (BCSC) considering clinical and demographic risk factors, a PRS, and moderate- and high-penetrance germline mutations [32]. WISDOM, the largest personalized screening trial in the USA, has demonstrated that population-based germline testing as part of breast cancer screening is both feasible and acceptable. Genetic testing is done once, can be less expensive than a mammogram and identifies women with the highest lifetime risk of cancer, and is a better way to assess the presence of an inherited mutation than family history [33]. The goal is to assign, assess, and improve screening guidance (when to start, how often to screen, and what modality to use) based on risk and breast density. The MyPeBS study in Europe is also testing risk-based screening in a study covering seven countries [34].

The heterogeneity of breast cancer is such that prevention and treatment strategies would ideally be tailored to the type of cancer likely to develop, but to accomplish this, we must upgrade our risk prediction tools. Most risk models today predict those who are at risk for slower growing, hormone-positive cancer, rather than faster growing cancers, because the former are the most common cancers [35, 36]. However, we need to improve our ability to screen for and intercept faster growing tumors, as they pose higher short-term mortality risk. WISDOM 2.0 is the next iteration of a personalized risk assessment and tailored screening and prevention, by integrating subtype-specific PRS scores as well as mammographic AI and assessing the impact of proposed changes in screening to the population at large. WISDOM 2.0 is open to women aged 30–75 without prior breast cancer diagnosis (https://www.thewisdomstudy.org/). Genetic testing can be done at any age. If we are going to look for mutation carriers, we should do so before they have the period of highest risk, which is in their 30s. We need better tools to predict who is at risk for what type of cancer, that are generalizable across all populations, which is a primary goal of WISDOM 2.0.

We also need to ensure models are generalizable to all populations, as most models currently have been developed on data obtained from women of European ancestry. Several groups are working on developing models that better predict who is at risk for what type of cancer across all racial and ethnic groups [37–39].

#### Provider education, the use of risk assessment tools in clinic, & standardized recommendations

A small percentage (estimated to be 18% in 2019) of OBGYNs, primary care doctors, and radiologists (the primary providers who provide breast cancer screening recommendations) currently use risk assessment tools in their practice [37–39]. There are no standardized recommendations of which model to use, which can lead to uncertainty [40]. Studies such as WISDOM are generating data using comprehensive risk assessment based on tumor type to determine if a standardized personalized risk assessment, screening and risk-reducing recommendations alters prevention choices and reduces risk. As well, these types of studies serve as templates for dissemination and integration of risk assessment for screening and prevention.

#### There are many available interventions that can reduce the risk of breast cancer

*Lifestyle interventions:* Minimizing alcohol, processed foods, exposure to toxins, stress/allostatic load, and increasing activity, muscle mass, and more have been shown to improve. menstrual symptoms, fertility, and breast cancer risk. A high BMI after menopause [41] and alcohol consumption [42] are both significant breast cancer risk factors. High-fiber diets may improve immunity and could be investigated as a risk reduction strategy in women at increased risk for immune-driven cancers [43] Regular exercise from childhood and adolescence can delay the onset of menarche, which can reduce breast cancer risk. Strategies to reduce BMI, including GLP-1 s are being explored as risk-reducing strategies [44, 45].

*Endocrine modulation—the challenge and promise*: Prophylactic use of selective estrogen receptor modulators (SERMs) like tamoxifen has been the gold standard in high-risk women [46, 47] since 1999. Aromatase inhibitors also decrease risk of HR-positive tumors by half [48, 49]. These medications reduce risk by about 50% for those at risk for HR-positive tumors and will be especially effective among those who are at high risk by high PRS [27] which predicts for slower growing HR-positive tumors.

However, despite large, robust trials demonstrating the benefits, SERMs and AIs are under-prescribed by primary care and gynecologic providers and have a very low uptake rate (5–10%) for high-risk women offered these medications [50], largely because of menopause-like side effects [51]. To pursue years of medication, women want to know that they are specifically at risk, that they would specifically benefit, and that they will not experience deleterious side effects.

Conditions like atypical ductal hyperplasia predict benefit from tamoxifen. Benefits continue to accrue for up to 10 years from 5 years of therapy. An early indicator that tamoxifen is working is the reduction of breast density [52, 53]. However, while the tools for measuring breast density exist, they are not readily clinically avaliable to measure treatment effect. The avaliability of mammographic AI will hopefully become more avaliable, but more research needs to be done to identify modifable markers that give women confidence that the medications they are taking are helping them specifically. Raloxifene (Evista) is a SERM that was developed to treat postmenopausal osteoporosis without causing an increased risk of endometrial cancer and was found to reduce breast cancer risk [54]. Raloxifene, like tamoxifen, increases hot flashes in about 15–20% of patients. In the long term, raloxifene is not as risk reducing as tamoxifen, but was thought to be safer in postmenopausal women. However, we now have a new alternative.

*BabyTam overcomes barriers to taking Tamoxifen*: Data now clearly show that lower doses of tamoxifen are as effective and well tolerated in the high-risk setting. A double-blind study, using mammographic breast density as a biomarker to predict benefit of tam, determined that doses as low as 2.5 mg of tamoxifen daily reduced density and that the lower doses were significantly better tolerated [55]. 5 mg of tamoxifen, when taken for 3 years, has been shown to reduce side effects, especially endometrial changes, and blood clots, are well tolerated and confer increasing risk reduction for up to 10 years [56]. “BabyTam,” can be given to both pre- and postmenopausal women safely with much improved uptake and confers the same cancer prevention benefit as full dose tamoxifen.

*Prophylactic Surgery*: For the very small population of women with lifetime risk of breast cancer that is over 60%, and especially where we have few risk-reducing options (e.g., BRCA1 mutation carriers who are at risk for HR-negative tumors and ovarian cancer), prophylactic mastectomy dramatically reduces the risk of getting and dying of cancer. Women who grew up in families where many family members died of breast and ovarian cancer are particularly motivated to pursue prophylaxis. If we develop agent combinations like PARP inhibitors that are highly effective for for treatment of BRCA1 tumors, we will likely be able to reduce the use of prophylactic surgery. Perhaps more important for mutation carriers, after childbearing is complete, is prophylactic removal of the ovaries and tubes to reduce the risk of ovarian cancer, especially because ovarian screening tools are ineffective. When done prior to 40, this will also reduce breast cancer risk [57, 58].

### Section 3: opportunities to incorporate breast cancer risk reduction into hormonal management across a woman’s lifetime

Optimizing prevention strategies requires an understanding of the types of breast cancer one is most likely to develop. Because 80% of breast cancers in the developed world are hormone driven [59], most current risk assessment algorithms (generated primarily from European populations in developed countries) predict the development of slower growing hormone-positive cancers [35, 39]. As we learn more about drivers of all cancer types, we will better understand the role that hormone modulation, for cycle control, fertility prevention, promotion, or preservation, and menopause symptom management  could play in reducing cancer risk.

Over the course of a woman’s lifetime, there are many times when hormonal manipulation is considered and prescribed. Redesigning common hormonal interventions with the goal of incorporating breast cancer risk reduction could shift the paradigm. Some of this work is already in progress.

#### Applying epidemiological insight about breast cancer risk surrounding hormone exposure

Mechanisms to reduce breast cancer risk could be integrated into the product design of hormonal agents used today. For the last 40 years, we have understood hormonal stimulation of breast tissue as one of the critical factors in breast cancer pathogenesis [60]. This likely explains why earlier menarche, later age at first pregnancy, later menopause, and higher total number of menstrual cycles over a life time are all associated with higher risk [57, 61]. A recent study provides a biologic underpinning for why risk is associated with each additional ovulatory cycle, where bouts of growth and regression drive the local and coordinated expansion and loss of mammary stem cells. While there are protective mechanisms to eliminate mutant clones that arise, including those that would drive a breast cancer, there is some chance of survival of mutant clones with each cycle, which could expand exponentially [58]. Additionally, suppression of the estrous cycle significantly limits the spread of mutant clones, even over the long term, which may explain why oophorectomy reduces the risk of BRCA1 breast tumors (which are largely hormone negative) to such a large extent in  premenopausal women [58]. Indeed, understanding this biology may help to inform the way we develop interventions for cycle control, contraception, and hormone replacement.

#### Lessons from the women’s health initiative (WHI)—the role of progestogens

The WHI was a landmark randomized controlled trial designed to study whether hormone replacement therapy (oral conjugated equine estrogens (CEE) with or without medroxyprogesterone acetate (MPA) should be used to prevent disease. When the trial detected a breast cancer incidence hazard ratio of 1.26, or absolute risk increase of 8 breast cancers in 10,000 women per year using combined hormone replacement therapy (as well as 8 more strokes, 8 more PEs, and 6 fewer colerectal cancers), the trial was stopped early [1, 62]. Of note, for those without a uterus taking CEE alone, breast cancer incidence decreased. Beyond the breast cancer findings, the study showed risk of venous thrombosis and cerebrovascular accident were increased for those taking combination or CEE alone. Following publication of the WHI results, there was a precipitous drop in prescriptions of combined hormone replacement, followed by one of the only declines in the incidence of breast cancer over the last 30 + years [63]. During this time, decades of WHI follow-up, re-analysis with age stratification, and several high quality but smaller randomized trials of newer formulations of hormones have revealed that the type, route, and timing of hormones have considerable impact on cardiovascular and cancer risks [62]. It is now recognized that oral estrogens and synthetic progestins are thrombogenic, while low-dose transdermal estradiol and oral micronized progesterone are not [62].

Importantly, while the MPA used in the WHI increased breast cancer incidence, not all progestogens do, either alone or in combination with estrogens although this is controversial. Small studies suggest that progesterone itself, unlike certain progestins, may not impact breast cancer risk. This would be especially good news, as it not only protects the endometrium but also treats insomnia and offers anxiolysis via its metabolite allopregnanolone-binding GABA-A receptors [62]. Drospirenone (DSP), which may be used for contraception and menopause symptom management, may also not increase risk [64].

Digging further into the question of how hormones impact breast health, cycled versus continuous hormones could have differential impact. We know that women with fewer menstrual cycles have less chance of breast cancer and cycling hormones may contribute to cancer development. Combined hormonal contraception effectively suppresses ovulation, but it is not clear that it is associated with a reduction in breast cancer risk. A recent review of hormonal contraception methods, including progestin-only methods, revealed an association with elevated risk [65]. More research is needed to tease out these associations further.

In addition to estrogen and progesterone,  androgen receptors appear to play a role in breast cancer, particularly in luminal B and rarer breast tumor types (neuroendocrine & lobular) that are less responsive to treatments. Efforts are underway to study androgen agonism (low-dose testosterone) as strategies for breast cancer prevention, focusing on reducing breast density and MRI contrast enhancement as modifiable risk factors (RECAST DCIS NCT06075953).

**Additional Opportunities**: While the details are beyond the scope of this paper, the following areas are ripe for rethinking opportunities to improve our interventions in the areas of cycle control, family planning/contraception, postpartum weaning, menopausal symptom management, in vitro fertilization (IVF), and transgender care. These are described in Table 1.

**Table 1:** Opportunities across women’s life course to achieve hormonal management and breast cancer risk reduction

Opportunity	Interventions	Relevance to breast cancer development and prevention	Opportunities to intervene to reduce breast cancer risk
Cycle Control	Reproductive-aged women may use hormonal methods not related to pregnancy prevention to help regulate their cycles for conditions like abnormal uterine bleeding, acne, or pelvic pain due to endometriosis or hemorrhagic cysts	Breast cancer risk has been linked to the total number of cycles and an early age of menarche [60]	Delaying the onset of menarche Reducing total number of cycles
Family planning/contraception	Oral contraceptive pills (OCPs) are prescribed to control ovulation and to time and prevent pregnancy 65% of women in the USA use some form of contraception. OCPs are one of the most common forms [68].	Possible increase in breast & cervical cancer rates with OCPs [69–72] Decrease in endometrial, ovarian, and colorectal cancers IUDs decrease endometrial shedding but do not impact number of menstrual cycles	Birth control that reduces the number of menstrual cycles Birth control that uses hormone formulations which may reduce breast cancer risk (e.g., continuous dosing, possibly drospirenone)
Postpartum Weaning	Postpartum involution of the breast (occurring after childbirth if lactation does not occur and after lactation stops with postpartum weaning) is when the breast tissue returns to its pre-pregnancy state. The microenvironment of postpartum involution is sufficient to promote breast cancer and its risk for metastasis and death	Pregnancy and breast feeding have been shown to have a protective effect against breast cancer incidence [72] Breast cancer diagnosed in the 5–10-year postpartum window is associated with more aggressive breast cancers with greater metastatic potential [72].	Targeting the involution period to significantly reduce the incidence or possibly the metastatic risk of breast cancer significantly [73] Using postpartum breast cancer as a model system to identify novel pathways and develop novel targeted therapy for breast cancer
Menopausal symptom management	Menopausal symptoms are caused by hormonal changes; in perimenopause, estrogen levels may be at times higher and other times lower than ever before, while progesterone declines through perimenopause. Symptoms may be caused by fluctuating levels and, as the transition progresses, increasingly by low estrogen and progesterone levels Menopausal symptoms impact over 80% of women lifetime & are debilitating for more than 25% [75] Menopausal hormone therapy is prescribed to ameliorate symptoms, and typically includes an estrogen and for those with a uterus, progestogen. It may be initiated in perimenopause or postmenopause	Combined hormone replacement therapies (estrogen + progestogen) have been shown to increase breast cancer risk [74] There is insufficient research to date, however small studies suggest certain progestogens may not be tumorigenic. Estrogen-alone therapy with CEE reduces risk, while estradiol alone may increase risk by a small amount [76] For women at high risk of breast cancer and cancer survivors, common forms of MHT could increase risk to intolerable levels. While there are non-hormonal treatments available to target each menopausal symptom, there is no strong singular approach that addresses the constellation of symptoms that would reliably be risk-neutral	For the genitourinary syndrome of menopause, most achieve substantial relief from local genital treatment with minimal systemic absorption. Some vaginal estrogen products are not associated with an increase in serum estradiol (estring, vagifem), and do not increase risk, while trials have shown testosterone cream is also beneficial and safe particularly in survivors of breast cancer For those requiring systemic therapy, SERDs and SERMs could help achieve all desired aims; because trials are costly and time-consuming, we must develop surrogate endpoints to expedite discovery Combination of low-dose estrogen + SERMs may ameliorate menopausal symptoms *and could be* breast cancer risk reducing. For example, bazedoxifene, a 4th-generation SERM with low-dose estradiol is being investigated for risk reduction in investigator-initiated studies [77]
In-vitro fertilization (IVF)	Egg harvesting requires injections of hormones to increase ovulation Techniques for fertility preservation have improved success and decreased costs Egg and embryo freezing are now both strongly viable options Making it more practical for more women to undergo egg retrieval to preserve fertility IVF also requires increased hormonal exposure	IVF exposes women to higher levels of hormones, increased ovulation Increasingly, women are opting to have children later in life and undergo egg harvesting or IVF There are some data to suggest that there is no significant association with breast cancer risk and use of IVF [77, 78]	Using aromatase inhibitors for egg harvesting is standard for women with a new diagnosis of breast cancer undergoing egg harvesting prior to starting chemotherapy harvesting [76]. This practice could be considered for all women undergoing IVF
Transgender Care	Testosterone is often taken as masculinizing therapy to affirm gender	Accumulating data show that testosterone is inhibits ductal growth with testosterone exposure, as evidence in mastectomy specimens from female to male transgender surgery [77, 78]. Testosterone is a potent inhibitor of the estrogen receptor Efforts are underway to study androgen agonism (low-dose testosterone) as a strategy for breast cancer prevention	Low dose testosterone plus AI is being investigated in the RECAST DCIS study to determine if it can be used in an active surveillance setting to reduce risk of DCIS (NCT06075953)

#### What will it take to develop and test these new approaches?

We need to use our imagination and scientific expertise to design better products, using early endpoints to predict risk, with registries to verify findings from trials and everyday use. To facilitate investment in the development of these new products, we will need to invest in:

1. **Intermediate endpoints that could short circuit the time to evaluate new agents or those already in use for other indications.** Prevention studies can be formidable because they take years to conduct and are enormously expensive [54]. To better evaluate drugs that are risk reducing for breast cancer, we need better mutable and modifiable biomarkers of risk to test these prevention opportunities in a shorter time frame—in the way that hypertension and cholesterol levels have become key biomarkers of risk for heart disease. Background parenchymal enhancement (BPE) measured on MRI [30] and breast density could have a similar utility in the setting of breast cancer. These endpoints found through imaging have been proposed and used as modifiable risk factors in several trials [55, 66, 79] and can be measured to analyze short-term impact and facilitate rapid learning, reducing the time and cost to evaluate these new agents.
2. **Regulatory strategies that will facilitate progress.** Companies with products for healthy women are reticent to investigate an association with cancer. There is also a reticence to investigate products that involve reproductive health, given the complex political climate surrounding this topic. It is unlikely that companies will invest in the time and effort to show a secondary indication for their drug unless there is a compelling reason to do so. We may need novel strategies for stimulating investment in this area, including philanthropy (Gates, 2024).

### Section 4: push to improve endocrine risk-reducing agents that are better tolerated & make our approach to hormonal management more nuanced, especially for survivors of breast cancer

The advances in prevention can also help us to provide better options for survivors of breast cancer, both for those in active treatment and years after diagnosis. There are millions of survivors of breast cancer, and for most survivors, endocrine therapy is the mainstay of treatment. Recurrence risk is often long in future after their first diagnosis and survivors are recommended to stay on these medications for years. However, many patients discontinue treatment (30% or more of patients discontinue this treatment after the first year), which raises recurrence risk [80]. For survivors who remain on treatment for 5 or 10 years of endocrine risk-reducing therapy, many struggle with hot flashes or osteoporosis, or other debilitating side effects, and frequently choose to discontinue therapy. There are several agents that are being studied that are more tolerable (selective estrogen receptor degraders). The same drugs we are trying to develop in the prevention setting so that they will be tolerable, improve quality of life AND be risk reducing for breast cancer could absolutely be used in the survivorship setting as well.

For many years, oncologists told women that it was dangerous to get pregnant for 5 years after a diagnosis of breast cancer until studies showed that there was no increased risk associated with pregnancy after a cancer diagnosis [81]. Further data obtained from the POSITIVE clinical trial confirmed these results [82]. Interestingly, estetrol, an ingredient in a newer OCP, described earlier, is made in high concentration in the placenta and may account for why pregnancy after breast cancer is not associated with an increased risk of recurrence. There are products that are safe for women after a diagnosis of HR-positive breast cancer (e.g., estring, vagifem) that do not elevate serum estrogen but reduce vaginal dryness and dyspareunia and frequent urinary tract infections. In clinical medicine, there are always trade-offs. A black and white approach can be replaced with an evidence-based, nuanced strategy to decision-making surrounding hormonal management by better understanding the type of cancer the period of risk, and degree of risk a woman is facing. However, we must generate the clinical data to inform decision-making.

### Section 5: work to reduce misinformation about hormonal products

Patients are increasingly turning to social media to supplement the information they receive from their care teams, often regarding the safety of hormonal intervention products, especially contraception [83–85]. Social media platforms like Instagram, TikTok, and YouTube can be effective vehicles for patient education [86–90]; however, the evidence-based information coming from reliable sources is often drowned out by misinformation that proliferates online [91, 92]. Misinformation content is constantly evolving. It often promotes supplements and products that are not FDA-approved above those that are. Compound formulations are common, but at this point should not be used because of poor data and quality control. In an FDA study of 29 compounded hormone therapy samples ordered over the internet, 34% did not meet one or more FDA quality standards, compared with a 2% failure rate among approved therapies [62]. The interplay of hormonal contraception and cancer risk is complicated and has changed over time as new formulations of contraception have come into use. On social media—which generally favors short-form content that makes nuance difficult to explain—non-biomedical influencers will often overstate this cancer risk, short-circuiting a good-faith discussion of the harms and benefits.

Related to the over-simplified statement that “birth control is a carcinogen” is the idea that preventing ovulation is overall detrimental to both short- and long-term health, even though these hormonal manipulations can empower women to have control over their reproductive health, manage significant menstrual and menopause symptoms, and could hold promise for breast cancer risk reduction in the future.

With 80% of women experiencing menopause symptoms at some point of their lives for an average of 4–7 years and very few clinicians aware of the latest society guidelines for thoughtful and safe prescription of menopausal therapies, it is no surprise women seek their own answers via the internet and as a result are susceptible to misinformation. Women deserve better.

Existing hormonal interventions, while not yet optimized in ways highlighted above, do offer great value nevertheless, so patients are harmed by the kind of misinformation that tells them to reject these and turn first to alternatives [93–95]. As this revolution in breast cancer care risk reduction unfolds, social media holds great promise to educate patients about the value of hormonal treatments optimized for cancer reduction as a component of holistic women’s healthcare. The first steps are to educate providers across disciplines, to counter misinformation and to encourage ongoing studies to constantly improve and refine information delivery.

### Section 6: to achieve this path forward, an integrated approach among all women’s health providers is critical, representing all populations

To achieve this paradigm shift and reduce misinformation surrounding hormonal products, both among providers and patients, we need to better integrate breast cancer risk assessment and reduction into primary care and OBGYN practice. At the same time, we must better equip breast oncologists with knowledge about hormonal management in women to better modulate and manage the side effects and hormonal transitions of patients who consider long-term recurrence risk-reducing endocrine therapy. Integrated training programs among women’s health providers that emphasize these principles (which currently exist at University of Michigan, The Cleveland Clinic, Mayo Clinic, and The University of California, San Francisco), and interdisciplinary conferences will help make this vision a reality. This progress needs to be made across clinics, from academic medical centers to community clinics, to FQHCs so all women benefit from these advances. That is the purpose of the RISE UP for Breast Cancer, a conference that convened for the first time in November 2024 (https://riseup.ucsf.edu/).

In conjunction, policies across the USA that restrict access to reproductive services create enormous issues of inequity and impact the ability of women’s health providers to provide vital care to women [96]. Full reproductive access is paramount to providing this holistic care to women. As champions for women’s health, physicians must advocate universal access to these important services. Part of this advocacy includes holding women’s health meetings in states that honor full reproductive care access [96–98].

There is much to learn and opportunity to think creatively to develop agents that serve more than one purpose. We need to press for funding and studies to explore new ideas, to develop agents that are health promoting, breast cancer risk reducing and tolerable for women. Importantly, these trials and innovations need to integrate women of all races and socio-economic status to make these findings fully generalizable. The proliferation of unfiltered and often misleading information about hormonal interventions, and the ubiquitous access through the internet makes it imperative that we in the medical community take these issues seriously and work to generate data that will help inform our patients.

A good place to start is beginning another series of well-designed prospective studies that include the NIH and the FDA to shed more light on the potential harms and substantial benefits that hormonal therapies can bring. We have learned enough that we should be testing agents designed explicitly to reduce breast cancer risk and introduce early endpoints to measure effectiveness, rather than to simply say that no one with high risk or history of breast cancer is eligible for symptom-reducing medications. Given that so many of the hormonal medications in use today have changed and long-term data is lacking, we should work to rethink and redesign interventions that are also risk-reducing. This could also provide options for symptom amelioration for all women, including those who are at high risk or have a prior history of breast cancer. This is a critical imperative that we should embrace.

## Conclusion

A paradigm shift in women’s health care that optimizes for reproductive autonomy, symptom management through pre-, peri-, and postmenopausal phases and actively pursues breast cancer prevention across the life course is possible and integral to driving down the persistently high rate of breast cancer. A concerted, integrated effort on breast cancer risk reduction could make an enormous impact on reducing the incidence of breast cancer. Tools exist to identify high-risk individuals, and there are ongoing studies to improve the prediction of who is at risk for what type of cancer. This will hopefully lead to tailored interventions for both screening and prevention. An important opportunity could lie in the redesign of drugs used for hormonal control across a woman’s lifetime. We should aspire to make routine hormonal interventions that reduce the risk of incident and recurrent breast cancer. Such products would have the potential to optimize for breast cancer prevention and improve the quality of life of survivors and maybe further reduce the risk of recurrence. This is a cause with potentially profound impact and is too tantalizing an opportunity for us not to embrace. This is the purpose of the new RISE UP (Revolutionizing Investigations to Step-Up Prevention) for Breast Cancer meeting, which debuted November 1–3 of 2024 in San Francisco (https://riseup.ucsf.edu/).

## Disclosures

DY reports research funding from Boehringer Ingelheim and Fusion Pharmaceuticals and is a consultant for Martell Diagnostics. CH reports institutional research funding from AstraZeneca, Gilead, Seagen/Pfizer, and Olema; individual consulting fees for running ad boards, moderating lectures, speaking/presenting fees from AstraZeneca, Seagen/Pfizer, Gilead, and Olema; and travel support from Olema. JG is an executive employee of Dana Faber Cancer Institute; participation on Fiduciary Board at American Association for Cancer Research; and participation on the Scientific Advisory Board at The James P. Wilmot Foundation, Inc. and Earli Inc. LE is a member of the Medical Advisory Panel for Blue Cross, Blue Shield; personal fees from UpToDate; and unpaid board member of QLHC. All other authors declare no competing interests.

## Data Availability

No datasets were generated or analysed during the current study.
