# Correction: A roadmap to reduce the incidence and mortality of breast cancer by rethinking our approach to women’s health

**DOI:** 10.1007/s10549-024-07572-8

**Published:** 2024-12-20

**Authors:** Katherine Leggat-Barr, Douglas Yee, Erin Duralde, Caroline Hodge, Virginia Borges, Molly Baxter, Jessica Valdez, Tamandra Morgan, Judy Garber, Laura Esserman

**Affiliations:** 1 Tufts Medical School, Boston, MA USA; 2 Masonic Cancer Center Minneapolis, University of Minnesota, Minneapolis, MN USA; 3 One Medical, Los Angeles, CA USA; 4 Department of Surgery, University of California, San Francisco, San Francisco, CA USA; 5 Division of Medical Oncology, University of Colorado Cancer Center, Aurora, CO USA; 6 Johns Hopkins Medical School, Baltimore, MD USA; 7 Dana Farber Cancer Institute, Boston, MA USA; 8 Alfred A de Lorimier Endowed Chair in General Surgery, 1825 4th St, 3rd Floor, San Francisco, CA 94158 USA

**Correction to: Breast Cancer Research and Treatment** 10.1007/s10549-024-07522-4

In this article, the following errors have been missed in the corrections stage and the same has been corrected with the correction article.

In the abstract section, the second sentence was incorrectly written as “Most women survive, but over 40,000 women a year still die of their disease National Cancer Institute [Internet]. [cited 2024 Nov 4]. Cancer of the Breast (Female)—Cancer Stat Facts. Available from: https://seer.cancer.gov/statfacts/html/breast.html” but should read as “Most women survive, but over 40,000 women a year still die of their disease (99)”.

In Section 2, 5th paragraph, 4th sentence, the hyperlink is incorrect. The correct sentence should read as “WISDOM 2.0 is open to women aged 30–75 without prior breast cancer diagnosis (https://thewisdomstudy.org).”

In Section 6, 1st paragraph, last sentence is incorrectly written as “That is the purpose of the RISE UP for Breast Cancer, a conference that will be convened for the first time in November 2024 (https://www.riseup.ucsf.edu).” but should read as "That is the purpose of RISE UP for Breast Cancer, a conference that convened for the first time in November 2024 (https://riseup.ucsf.edu).”

In the last line of conclusion section, hyperlink was missing for the text “riseup.ucsf.edu”. The correct text should be “https://riseup.ucsf.edu”.

The following reference has been included in the reference list: National Cancer Institute [Internet]. [cited 2024 Nov 4]. Cancer of the Breast (Female)—Cancer Stat Facts. Available from: https://seer.cancer.gov/statfacts/html/breast.html

The original article has been corrected.

